# Association of glycemic variability with oxidative stress and AGE accumulation in type 2 diabetes

**DOI:** 10.1038/s41598-025-31845-x

**Published:** 2025-12-11

**Authors:** Makoto Ohara, Noriyuki Takahashi, Nobuaki Takehana, Naoya Osaka, Hiroe Sugita, Michishige Terasaki, Yusaku Mori, Tomoyasu Fukui, Sho-ichi Yamagishi

**Affiliations:** 1 Department of Medicine, Division of Diabetes, Metabolism, and Endocrinology, Showa Medical University Graduate School of Medicine, 1-5-8 Hatanodai, Shinagawa-Ku, Tokyo, 142-8666 Japan; 2 Department of Medicine, Division of Diabetes, Metabolism, and Endocrinology, Antiglycation Research Section, Showa Medical University Graduate School of Medicine, Tokyo, Japan

**Keywords:** Biomarkers, Diseases, Endocrinology, Medical research, Nephrology

## Abstract

Oxidative stress and advanced glycation end products (AGEs) could promote vascular aging in type 2 diabetes mellitus (T2DM), but their relationship with glycemic variability remains unclear. We conducted a cross-sectional study of 68 patients with T2DM who underwent continuous glucose monitoring (CGM). Short-, intermediate-, and long-term glycemic variability were assessed using the % coefficient of variation of glucose (%CV), mean of daily differences (MODD), and HbA1c variability over the prior 2 years, respectively. Oxidative stress was measured by the diacron-reactive oxygen metabolites (d-ROMs) test, and AGE accumulation by skin autofluorescence (SAF). Multivariate analyses identified female sex, %CV, MODD, and nephropathy stage as independent correlates of d-ROMs (adjusted R^2^ = 0.436). Duration of diabetes, MODD, urinary albumin-to-creatinine ratio, and macroangiopathy were independently associated with SAF (adjusted R^2^ = 0.265). This study suggests that short- and intermediate-term glycemic variability correlate with oxidative stress and AGE accumulation, which may be associated with vascular complications in T2DM.

**Supplementary Information:**

The online version contains supplementary material available at 10.1038/s41598-025-31845-x.

## Introduction

Type 2 diabetes mellitus (T2DM) is increasingly recognized as a model of accelerated aging, which could markedly increase the risk of multiple age-related diseases, such as cardiovascular disease (CVD), chronic kidney disease, and cognitive decline^1–3^. Shared pathophysiological features between T2DM and biological aging include chronic inflammation, mitochondrial dysfunction, oxidative stress, and the accumulation of advanced glycation end products (AGEs)^1,4–7^. These processes are thought to link T2DM to the development of various age-related complications^1,4,6,7^. However, the interrelationships among glycemic variability, oxidative stress, and AGE accumulation remain poorly understood.

Glycated hemoglobin (HbA1c) is the gold standard marker of glycemic management for patients with T2DM and predicts the risk of various diabetes-related complications in these patients^2,3,8,9^. However, HbA1c reflects only average glucose levels over the preceding 2–3 months and does not capture short-term glycemic variability or long-term cumulative glycemic burden, including postprandial hyperglycemia and hypoglycemia^10^. On the other hand, glycemic variability has also been recognized as a contributor to oxidative stress, endothelial dysfunction, and vascular complications in T2DM^11–13^. While the United Kingdom Prospective Diabetes Study (UKPDS) demonstrated that early intensive glycemic control reduced microvascular complications and long-term cardiovascular risk in T2DM patients^14,15^, other clinical trials showed that intensive HbA1c lowering did not necessarily reduce cardiovascular events^16,17^. These findings could highlight the limitations of relying solely on HbA1c to assess macrovascular risk and underscore the importance of evaluating other glycemic control markers, such as glycemic variability.

Oxidative stress could be a key contributor to the progression of vascular complications in T2DM^8,18,19^. A large-scale cohort study found that levels of diacron-reactive oxygen metabolites (d-ROMs), which serve as a biomarker of reactive oxygen species (ROS) production, were significantly associated with major cardiovascular events in patients with T2DM^19^. Furthermore, chronic hyperglycemia promotes the formation and accumulation of AGEs, which could trigger ROS generation and inflammatory reactions in T2DM^12,20^. Recent technological advances have enabled a non-invasive assessment of AGE accumulation in the human body, which was evaluated as skin autofluorescence (SAF) using the AGE-Reader (Diagnoptics, Groningen, Netherlands)^21^. Indeed, SAF has been associated with various aging-related complications in patients with diabetes^22–24^.

Despite these observations, few studies have simultaneously evaluated glycemic variability, oxidative stress, and AGE accumulation within the same cohort. Understanding their interrelationships may provide new insights into the shared mechanisms underlying diabetic vascular complications and vascular aging. Therefore, we hypothesized that glycemic variability was associated with increased oxidative stress and AGE accumulation, which may be involved in the pathophysiology of diabetic vascular complications. To test this hypothesis, we investigated the associations of short-, intermediate-, and long-term glycemic variability with oxidative stress and AGE accumulation in patients with T2DM.

## Results

### Patient sample and characteristics

Table 1 shows the participants’ clinical and biochemical characteristics, while Table 2 illustrates the details regarding medications for T2DM, hypertension, and dyslipidemia. The study included a total of 68 patients (39 males and 29 females) with a mean age of 68.5 ± 10.6 years. The median duration of diabetes was 12.0 (6.0–20.0) years. The mean HbA1c was 7.5% ± 0.9%, while the 2-year average HbA1c was 7.4% ± 0.3%. Hypertension and dyslipidemia were present in 70.6% and 88.2% of patients, respectively. At baseline, the medications for T2DM included metformin (45.6%), glucagon-like peptide-1 receptor agonists (39.7%), sodium-glucose co-transporter 2 inhibitors (36.8%), insulin (33.8%), and dipeptidyl peptidase-4 inhibitors (32.4%).

**Table 1:** Baseline clinical characteristics of subjects.

Clinical characteristics	Means ± SD, n (%)
Age (years)	68.5 ± 10.6
Sex (male)	39 (57.4)
Body mass index (kg/m^2^)	25.4 ± 4.0
Smoking (%)	3 (4.4)
Duration of diabetes (years)	12.0 (6.0–20.0)
Hypertension	48 (70.6)
Dyslipidemia	60 (88.2)
Blood pressure (mmHg)	
Systolic	136.8 ± 18.5
Diastolic	76.2 ± 12.3
Low-density lipoprotein cholesterol (mg/dL)	88.0 ± 23.7
High-density lipoprotein cholesterol (mg/dL)	53.9 ± 21.5
Triglycerides (mg/dL)	106.0 (75.5–143.5)
Estimated glomerular filtration rate (ml/min/1.73 m^2^)	71.5 ± 19.0
Fasting plasma glucose state (mg/dL)	137.1 ± 32.0
Fasting c-peptide (ng/mL)	1.8 ± 0.9
HbA1c (%; mmol/mol)	7.5 ± 0.9 (59 ± 10)
CGM metrics	
Mean glucose level (mg/dL)	163.1 ± 34.4
Glucose management indicator (%)	7.2 ± 0.8
Markers of glucose variability	
%CV	24.4 ± 7.0
MAGE (mg/dL)	108.0 (92.4–133.8)
MODD (mg/dL)	32.3 (23.6–44.9)
Time in range (%)	74.3 (52.0–89.5)
Time above range (%)	25.3 (10.5–44.8)
Time below range (%)	0.0 (0.0–0.0)
Markers of long-term glucose variability	
HbA1c SD (%)	0.39 (0.26–0.49)
HbA1c CV (%)	5.3 (3.7–6.7)
2-year average HbA1c (%; mmol/mol)	7.4 ± 0.7 (57 ± 8)
UACR (mg/g・Cre)	28.7 (12.7–205.7)
d-ROMs (U.CARR)	349.4 ± 69.4
Skin autofluorescence (AU)	3.0 ± 0.6
Macroangiopathy	17 (25.0)
Neuropathy	43 (63.2)
Retinopathy (NDR/SDR/PPDR/PDR)	39/17/2/10
Nephropathy (normoalbuminuria/microalbminuria/macroalbuminuria)	32/22/14

Data are expressed as mean ± standard deviation, medians (interquartile ranges), or numbers (%).

*HbA1c* hemoglobin A1c,* CGM* continuous glucose monitoring,* %CV* coefficient of variation,* MAGE* mean amplitude of glycemic excursions,* MODD* mean of daily difference of blood glucose,* SD* standard deviation,* UACR* urinary albumin-to-creatinine ratio,* d-ROMs* diacron-reactive oxygen metabolites,* 1 U.CARR (arbitrary unit) * the oxidant capacity of a 0.08 mg/dL H_2_O_2_ solution.* NDR* no diabetic retinopathy,* SDR* simple diabetic retinopathy,* PPDR* pre-proliferative diabetic retinopathy,* PDR* proliferative diabetic retinopathy.

**Table 2:** Medications for diabetes, hypertension, and dyslipidemia.

Treatment	n (%)
Diabetes therapy	
Diet alone	6 (8.8)
Metformin	31 (45.6)
Sulfonylureas	6 (8.8)
Glinides	9 (13.2)
α-glucosidase inhibitors	21 (30.9)
Thiazolidine	5 (7.4)
Dipeptidyl peptidase 4 inhibitors	22 (32.4)
Sodium-glucose cotransporter 2 inhibitors	25 (36.8)
Glucagon-like peptide 1 receptor agonists	27 (39.7)
Insulins	23 (33.8)
Antihypertensive drugs	
Renin–angiotensin–aldosterone system inhibitors	38 (55.9)
Calcium channel blockers	26 (38.2)
Diuretics	4 (5.9)
α-Blockers	1 (1.5)
β-Blockers	8 (11.8)
Lipid-lowering drugs	
Statins	50 (73.5)
Ezetimibe	4 (5.9)
Fibrates	5 (7.4)

### Correlation of d-ROM levels with clinical and biochemical variables

Table 3 shows the correlations between d-ROMs levels and the clinical and biochemical variables. Significant correlations were observed between d-ROMs levels and HbA1c (r = 0.399, p = 0.001), mean glucose level (MGL) and glucose management indicator (GMI) (r = 0.319, p = 0.008), % coefficient of variation of glucose (CV) (r = 0.434, p < 0.001), mean amplitude of glycemic excursions (MAGE) (r_s_ = 0.523, p < 0.001), mean of daily differences (MODD) (r_s_ = 0.556, p < 0.001), time in range (TIR) (r_s_ =  − 0.349, p = 0.004), time above range (TAR) (r_s_ = 0.349, p = 0.001), HbA1c standard deviation (SD) (r_s_ = 0.382, p = 0.001), 2-year average HbA1c (r = 0.407, p = 0.001), and HbA1c CV (r_s_ = 0.316, p = 0.009). The d-ROMs levels significantly differed between the groups with and without hypertension (p = 0.012), thiazolidine use (p = 0.045), insulin use (p = 0.033), female sex (p = 0.021), and by diabetic nephropathy stage (p = 0.013). Multiple stepwise regression analysis was performed to evaluate the intercorrelations among these parameters, revealing that the following variables were independently correlated with d-ROMs levels (adjusted R^2^ = 0.436): female sex (β = 0.322, p = 0.001), %CV (β = 0.225, p = 0.042), MODD (β = 0.402, p = 0.001), and diabetic nephropathy stage (β = 0.202, p = 0.036). Full regression coefficients (B), standardized coefficients (β), t-values, variance inflation factors (VIF), and 95% confidence intervals are presented in Supplementary Table 1. As shown in Supplementary Table 1, GLP-1 receptor agonist and SGLT2 inhibitor use were also included as candidate variables in the stepwise multiple regression analysis, but were automatically excluded by the selection procedure.

**Table 3:** Correlations of clinical variables with d-ROMs.

Independent variables	Univariate		Multivariate	
	r/r_s_	P	β	P
Age	0.042	0.733		
Sex(Male:1、Female:2)	-	**0.021**	0.322	**0.001**
Body mass index (kg/m^2^)	-0.129	0.294		
Smoking	-	0.106		
Duration of diabetes (years)	0.114	0.355		
Hypertension	-	**0.012**		
Dyslipidemia	-	0.833		
Systolic blood pressure (mmHg)	0.108	0.380		
Diastolic blood pressure (mmHg)	0.098	0.424		
Low-density lipoprotein cholesterol (mg/dL)	0.042	0.733		
High-density lipoprotein cholesterol (mg/dL)	0.033	0.789		
Triglycerides (mg/dL)	0.062	0.617		
Estimated glomerular filtration rate (ml/min/1.73 ${\mathrm{m}}^{2}$)	0.004	0.977		
Fasting plasma glucose (mg/dL)	0.071	0.566		
Fasting C-peptide immunoreactivity (ng/mL)	-0.150	0.222		
HbA1c (%)	0.399	**0.001**		
Mean glucose level (mg/dL)	0.319	**0.008**		
Glucose management indicator (%)	0.319	**0.008**		
%CV	0.434	** < 0.001**	0.225	**0.042**
MAGE (mg/dL)	0.523	** < 0.001**		
MODD (mg/dL)	0.556	** < 0.001**	0.402	**0.001**
Time in range (%)	-0.349	**0.004**		
Time above range (%)	0.349	**0.001**		
Time below range (%)	0.234	0.055		
HbA1c SD (%)	0.382	**0.001**		
2-year average HbA1c (%)	0.407	**0.001**		
HbA1c CV (%)	0.316	**0.009**		
UACR (mg/g・Cre)	0.232	0.057		
Macroangiopathy	-	0.381		
Neuropathy	-	0.106		
Retinopathy	-	0.247		
Nephropathy	-	**0.013**	0.202	**0.036**
Diabetes therapy				
Diet alone	-	0.915		
Metformin	-	0.467		
Sulfonylureas	-	0.364		
Glinides	-	0.087		
α-glucosidase inhibitors	-	0.132		
Thiazolidine	-	**0.045**		
Dipeptidyl peptidase 4 inhibitors	-	0.693		
Sodium-glucose cotransporter 2 inhibitors	-	0.264		
Glucagon-like peptide 1 receptor agonists	-	0.702		
Insulins	-	**0.033**		
Antihypertensive drugs				
Renin–angiotensin–aldosterone system inhibitors	-	0.289		
Calcium channel blockers	-	0.053		
Diuretics	-	0.219		
α-Blockers	-	0.137		
β-Blockers	-	0.696		
Lipid-lowering drugs				
Statins	-	0.749		
Ezetimibe	-	0.939		
Fibrates	-	0.979		
${\mathrm{R}}^{2}$			0.436	

Data are expressed as correlation coefficients (r/rs) for univariate analyses and standardized regression coefficients (β) for multivariate analyses. Pearson’s or Spearman’s correlation coefficients were used as appropriate. Variables with P < 0.05 in univariate analysis were considered for multivariate linear regression analysis to identify independent correlates of oxidative stress measured by d-ROMs.

*HbA1c* hemoglobin A1c, *CGM* continuous glucose monitoring, *%CV* coefficient of variation, *MAGE* mean amplitude of glycemic excursions, *MODD* mean of daily difference of blood glucose, *SD* standard deviation, *UACR* urinary albumin-to-creatinine ratio, *d-ROMs* diacron-reactive oxygen metabolites, *1 U.CARR (arbitrary unit)* = the oxidant capacity of a 0.08 mg/dL H_2_O_2_ solution.

### Correlation of SAF with clinical and biochemical variables

Table 4 shows the correlations between SAF and the clinical and biochemical variables. Significant correlations were observed between SAF and age (r = 0.284, p = 0.019), duration of diabetes (r_s_ = 0.365, p = 0.002), low-density lipoprotein cholesterol (LDL-C) (r =  − 0.253, p = 0.037), estimated glomerular filtration rate (eGFR) (r =  − 0.247, p = 0.042), %CV (r = 0.243, p = 0.046), MODD (r_s_ = 0.246, p = 0.043), 2-year average HbA1c (r = 0.295, p = 0.015), and urinary albumin-to-creatinine ratio (UACR) (r_s_ = 0.261, p = 0.032). The SAF values significantly differed between the groups with and without hypertension (p = 0.003), dyslipidemia (p = 0.035), macroangiopathy (p = 0.005), neuropathy (p = 0.008), diet therapy (p = 0.001), insulin use (p = 0.021), diuretic use (p = 0.024), and diabetic retinopathy stage (p = 0.013). Multiple stepwise regression analysis revealed that the following variables were independently correlated with SAF (adjusted R^2^ = 0.265): duration of diabetes (β = 0.249, p = 0.029), MODD (β = 0.216, p = 0.049), UACR (β = 0.220, p = 0.032), and macroangiopathy (β = 0.272, p = 0.016). B, β, t-values, VIF, and 95% confidence intervals are presented in Supplementary Table 2. As shown in Supplementary Table 2, use of GLP-1 receptor agonists and SGLT2 inhibitors were also included as candidate variables in the stepwise multiple regression analysis, but were automatically excluded by the selection procedure.

**Table 4:** Correlations of clinical variables with SAF.

Independent variables	Univariate		Multivariate	
	r/r_s_	P	β	P
Age	0.284	**0.019**		
Sex	-	0.578		
Body mass index (kg/m^2^)	-0.082	0.507		
Smoking	-	0.447		
Duration of diabetes (years)	0.365	**0.002**	0.249	**0.029**
Hypertension	-	**0.003**		
Dyslipidemia	-	**0.035**		
Systolic blood pressure (mmHg)	-0.035	0.779		
Diastolic blood pressure (mmHg)	-0.187	0.127		
Low-density lipoprotein cholesterol (mg/dL)	-0.253	**0.037**		
High-density lipoprotein cholesterol (mg/dL)	-0.145	0.238		
Triglycerides (mg/dL)	-0.018	0.883		
Estimated glomerular filtration rate (ml/min/1.73 ${\mathrm{m}}^{2}$)	-0.247	**0.042**		
Fasting plasma glucose (mg/dL)	0.070	0.571		
Fasting C-peptide immunoreactivity (ng/mL)	-0.172	0.161		
HbA1c (%)	0.104	0.400		
Mean glucose level (mg/dL)	0.127	0.303		
Glucose management indicator (%)	0.127	0.303		
%CV	0.243	**0.046**		
MAGE (mg/dL)	0.134	0.275		
MODD (mg/dL)	0.246	**0.043**	0.216	**0.049**
Time in range (%)	-0.101	0.411		
Time above range (%)	0.105	0.393		
Time below range (%)	0.060	0.624		
HbA1c SD (%)	0.134	0.275		
2-year average HbA1c (%)	0.295	**0.015**		
HbA1c CV (%)	0.099	0.424		
UACR (mg/g・Cre)	0.261	**0.032**	0.220	**0.032**
Macroangiopathy	-	**0.005**	0.272	**0.016**
Neuropathy	-	**0.008**		
Retinopathy	-	**0.013**		
Nephropathy	-	0.166		
Diabetes therapy				
Diet alone	-	**0.001**		
Metformin	-	0.061		
Sulfonylureas	-	0.763		
Glinides	-	0.784		
α-glucosidase inhibitors	-	0.327		
Thiazolidine	-	0.891		
Dipeptidyl peptidase 4 inhibitors	-	0.799		
Sodium-glucose cotransporter 2 inhibitors	-	0.143		
Glucagon-like peptide 1 receptor agonists	-	0.227		
Insulins	-	**0.021**		
Antihypertensive drugs				
Renin–angiotensin–aldosterone system inhibitors	-	0.174		
Calcium channel blockers	-	0.912		
Diuretics	-	**0.024**		
α-Blockers	-	0.970		
β-Blockers	-	0.245		
Lipid-lowering drugs				
Statins	-	0.497		
Ezetimibe	-	0.248		
Fibrates	-	0.257		
${\mathrm{R}}^{2}$			0.265	

Data are expressed as correlation coefficients (r/rs) for univariate analyses and standardized regression coefficients (β) for multivariate analyses. Pearson’s or Spearman’s correlation coefficients were used as appropriate. Variables with P < 0.05 in univariate analysis were included in the multivariate linear regression analysis to determine independent correlates of skin autofluorescence (SAF), a surrogate marker of AGE accumulation.

*HbA1c* hemoglobin A1c, *CGM* continuous glucose monitoring, *%CV* coefficient of variation, *MAGE* mean amplitude of glycemic excursions, *MODD* mean of daily difference of blood glucose, *SD* standard deviation, *UACR* urinary albumin-to-creatinine ratio.

## Discussion

There is a growing body of evidence to show that d-ROMs are a clinically applicable biomarker of oxidative stress and have a potential clinical utility for predicting CVD events in patients with or without diabetes^19,25–30^. Indeed, elevated d-ROMs levels have been reported to be associated with the increased incidence of CVD events and all-cause mortality in the both general population and high-risk subjects^19,25,27–30^. Furthermore, a pooled analysis of two large cohorts of patients with T2DM demonstrated that higher d-ROMs levels were significantly associated with the risk of major adverse CVD events and all-cause death^19^. In our T2DM subjects, the mean d-ROMs value was 349.4 ± 69.4 U.CARR, which exceeded the normal reference range of 250–300 U.CARR^31^. Based on the previous cohort data^19^, the elevation in d-ROMs of our patients may correspond to an approximately 1.5–2.0-fold increased risk in CVD events compared with non-diabetic individuals. These findings suggest that our study participants were exposed to a substantial degree of oxidative stress, which may potentially place them at the elevated risk for CVD. In addition to chronic hyperglycemia, data from in vitro cell and in vivo animal experiments to clinical studies have suggested that glycemic variability could induce oxidative stress^31–33^. Fluctuating glucose levels have been shown to provoke mitochondrial ROS production more strongly than sustained chronic hyperglycemia^34^. Based on these backgrounds, the aim of the present study is to explore whether d-ROMs and SAF, markers of oxidative stress and cumulative hyperglycemic exposure, respectively, are associated with short-term (%CV and MAGE), intermediate-term (MODD), and long-term (HbA1c-CV and SD) glycemic variability in patients with T2DM.

In this study, we found that female sex, %CV, MODD, and diabetic nephropathy stage emerged as independent correlates of oxidative stress, as assessed by d-ROMs. The sex difference in d-ROMs observed here was consistent with the previous report showing that women tend to exhibit higher oxidative stress markers than men^35^. The mean age of our study participants was 68.5 years old, and most of the women were postmenopausal. Therefore, the decline in anti-oxidative effects of estrogen may partly explain the present result^36^. The independent positive associations of %CV and MODD, which could reflect short- and intermediate-term glycemic variability, respectively, with d-ROMs could reinforce the concept that intraday and day-to-day glycemic variability is closely linked to oxidative stress generation in patients with T2DM, and these results were consistent with our previous finding indicating that MAGE and MODD were correlated with d-ROMs in T2DM patients^31^. Notably, in the present study, %CV was a stronger and independent correlate of d-ROMs than MAGE, a traditional biomarker of short-term glycemic variability^37^. This may be explained in part by the difference in background of study participants between the present study and the previous one; our cohort consisted of relatively well-controlled T2DM patients (mean HbA1c: 7.4%), while participants of the previous study exhibited HbA1c values ≥ 8.0%^11,31,38^. Since %CV is calculated by dividing the SD by MGL, it may offer a more standardized and clinically relevant marker of intraday glycemic variability in such well-controlled T2DM patients. These findings align with and support current international recommendations that highlight %CV as a practical and clinically useful metric for evaluating the glycemic variability in routine diabetes care^39^. Furthermore, the finding that MODD was independently associated with d-ROMs suggests the potential clinical utility of measuring the day-to-day glycemic variability for evaluating future CVD risk in T2DM patients that may not be fully captured by other continuous glucose monitoring (CGM) metrics. In the present study, markers of long-term glycemic variability, such as CV or SD of HbA1c, were not independently associated with oxidative stress; they were only correlated with d-ROMs in the univariate analysis. The finding was in contrast with the previous study by Chang et al*.*, which showed that besides short-term glucose variability, HbA1c variability was associated with oxidative stress^38^. In their study, the mean HbA1c SD was 1.3 ± 0.6%, whereas ours was 0.39 (0.26–0.49) %. The difference in HbA1c SD between ours and theirs may partly account for the discrepant result. The independent correlation of d-ROMs with diabetic nephropathy stage observed here could further support the pathophysiological role of oxidative stress in the development and progression of diabetic nephropathy^40^; d-ROMs may also become a biomarker for predicting the risk of diabetic nephropathy.

In patients with T2DM, SAF levels around 3.0 arbitrary units (AU) have been linked to a 2–3-fold higher risk of CVD and mortality compared with non-diabetic subjects^41^. Therefore, our cohort (mean SAF: 3.0 ± 0.6 AU) represents a clinically high-risk population for CVD. AGE accumulation levels in the skin evaluated by SAF with an AGE-Reader have been reported to predict future cardiovascular events and death in T2DM patients^42^. Furthermore, many preclinical and clinical studies have suggested the pivotal role of AGEs in the pathogenesis of diabetic vascular complications^22–24,43^. Since the formation and accumulation of AGEs are stimulated by postprandial hyperglycemia, which in concert with AGEs could evoke oxidative stress generation^44,45^, we examined here the correlations of SAF with short-, intermediate-, and long-term glycemic variability and d-ROMs in patients with T2DM.

In this study, we found that duration of diabetes, MODD, UACR, and macroangiopathy were independent correlates of SAF. The association with duration of diabetes is plausible because SAF is a marker of AGE accumulation levels in the skin, which could reflect cumulative hyperglycemic exposure^21^. Although SAF was significantly correlated with the 2-year mean HbA1c in univariate analysis, the association was lost after multivariate adjustment. This may be due to the relatively short-term observation period for evaluating mean HbA1c levels in our study. In other words, 2-year mean HbA1c levels may not sufficiently capture long-term hyperglycemic exposure, which was a reason why mean HbA1c levels in our subjects were not independently correlated with SAF. Indeed, Rigo et al*.* investigated the association between SAF and mean HbA1c levels stratified by interval time of measurements for mean HbA1c values (short period: past 3 months, medium period: past 14 months, long period: past 30 months) and found a significant correlation was only observed between SAF and mean HbA1c values with a long interval time^46^. These findings suggest that sustained hyperglycemia for at least 2.5 years may be necessary for evaluating the AGE accumulation by SAF. In the present study, MODD was independently associated with SAF, while %CV, MAGE, and long-term HbA1c variability indices were not. We did not know the exact reason why day-to-day glycemic variability was independently correlated with AGE accumulation levels. However, we have previously shown that 3-month intervention of postprandial hyperglycemia by acarbose significantly decreases serum AGEs levels, but not HbA1c values in well-controlled T2DM patients (HbA1c levels: 6.5%)^44^. Therefore, intermediate-term glycemic variability evaluated by MODD may more strongly affect the AGE accumulation levels in well-controlled T2DM patients such as our cohort. In support of the clinical relevance of MODD, Miyoshi et al*.* recently demonstrated that increased day-to-day glycemic variability, as assessed by MODD, was a significant predictor of cardiovascular events in patients after acute coronary syndrome^47^. Moreover, the present findings that UACR and macroangiopathy were independently associated with SAF, support the pathological involvement of AGEs in diabetic nephropathy and CVD in T2DM patients^48^.

The novelty of our study lies in the simultaneous assessment of oxidative stress and AGE accumulation in relation to short-, intermediate-, and long-term glycemic variability using CGM. This integrative approach has not been previously reported in patients with T2DM. Furthermore, although this study was conducted in a Japanese cohort, the underlying mechanisms linking glycemic variability, oxidative stress, and AGE accumulation are based on fundamental biological processes that are not ethnicity-specific. Therefore, our findings may be generalizable to other populations, but further validation in multi-ethnic cohorts is warranted.

This study has several limitations. First, it employed a cross-sectional design without a control group, which prevents any assessment of causal relationships. Therefore, the associations observed here should be interpreted as hypothesis-generating. Further prospective research is needed to establish the causality. Second, the final sample size was smaller than initially planned because the iPro2 CGM system was discontinued during the study period, which limited further participant enrollment. This reduced sample size may have decreased the statistical power to detect subtle associations, particularly in subgroup analyses. However, in the multiple stepwise regression analyses, key glycemic variability indices remained statistically significant independent correlates of both oxidative stress and AGE accumulation. For oxidative stress (d-ROMs), %CV (β = 0.225, p = 0.042), MODD (β = 0.402, p = 0.001), female sex (β = 0.322, p = 0.001), and nephropathy stage (β = 0.202, p = 0.036) remained significant predictors, with all VIF values below 1.5, indicating no concerning multicollinearity. The adjusted R^2^ value of 0.436 further suggests that the model had a moderate explanatory capacity. In addition, duration of diabetes (β = 0.249, p = 0.029), MODD (β = 0.216, p = 0.049), UACR (β = 0.220, p = 0.032), and macroangiopathy (β = 0.272, p = 0.016) were independent correlates of AGE accumulation (SAF), with VIF values below 1.2 and an adjusted R^2^ value of 0.265, also indicating moderate explanatory power. Therefore, the present findings may still provide meaningful exploratory insights despite the limited number of participants. Given that the reduced sample size may limit the generalizability of the results, larger studies will be necessary to confirm these associations. Third, although we examined long-term glycemic variability using HbA1c data over a two-year period, this duration may not be sufficient to fully capture long-term glycemic variability, thus potentially underestimating its relationship with SAF. Fourth, the duration of hypertension and dyslipidemia, which could influence oxidative stress and AGE accumulation, was not evaluated in our analysis, which may confound the present results. Fifth, dietary habits and lifestyle factors, which have been reported to influence the SAF levels^49^, were not thoroughly assessed in this study. Future studies employing larger cohorts, longer observation periods, and comprehensive assessments of lifestyle factors are needed to validate and extend the present findings. Sixth, in this study, all participants wore the iPro2 (Medtronic MiniMed) CGM system for four consecutive days. Although current consensus guidelines recommend using at least 70% of CGM data collected over a 14-day period^39^, the glycemic variability indices (%CV, MAGE, and MODD) were calculated using only two full 24-h recordings (days 2 and 3) because day 1 included the initial calibration phase and day 4 sometimes lacked complete data. The MARD value of 8.0 ± 9.0% for the iPro2 originates from validation studies comparing CGM values with capillary glucose measurements; however, this value was not derived from a dedicated 2-day analytic window. To the best of our knowledge, no published study has reported a MARD value specifically calculated from a 2-day wear period of the iPro2 device. Therefore, we cannot directly confirm whether the MARD during a 2-day analysis would be below 10%. We fully acknowledge that the short, 2-day monitoring duration may not fully capture overall glycemic patterns, particularly day-to-day variability, and that the lack of MARD during a 2-day wear analysis could limit the validity of our findings.

In conclusion, this study suggests that short- and intermediate-term glycemic variability, oxidative stress, and AGE accumulation are interrelated and may be associated with nephropathy and macroangiopathy in patients with T2DM. These findings suggest that oxidative stress markers and SAF may serve as promising non-invasive indicators for identifying patients at higher risk of vascular complications in T2DM patients.

## Methods

### Study design

This single-center, cross-sectional study was approved by the Ethics Committee of Showa Medical University (approval number 2930), and written informed consent was obtained from all participants. This study was registered in the University Hospital Medical Information Network Clinical Trials Registry (ID: UMIN000041818) and conducted in accordance with the Declaration of Helsinki and its subsequent revisions.

### Subjects

Between October 2019 and December 2021, this study enrolled 68 outpatients with T2DM who had been receiving care at Showa Medical University Hospital for at least 2 years. Based on previously published studies that evaluated glycemic variability and oxidative stress in patients with T2DM using CGM systems^11,31,38^, we deemed that a sample size of 100 outpatients was appropriate for an exploratory analysis without a formal power calculation. However, due to the unexpected discontinuation of the iPro2 CGM device service (the CGM system used throughout this study), data collection was halted at 68 cases. This study included patients aged at least 20 years old and diagnosed with T2DM. The exclusion criteria were as follows: current use of steroids or anti-inflammatory medications; diabetic ketoacidosis or coma within 3 months prior to study initiation; current severe infection, major trauma, or perioperative status; eGFR of ≤ 30 mL/min/1.73 m^2^; significant hepatic dysfunction; known malignancy; pregnancy; or any condition deemed inappropriate for study participation by the treating physician.

### Procedures and measurements

**Fig. 1:**
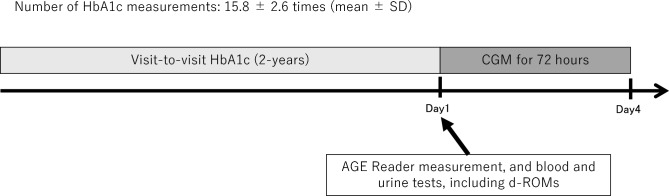
Overview of the study protocol.

All participants were equipped with a CGM device (iPro2, Medtronic MiniMed, Northridge, CA, USA) for 4 days. The CGM metrics, including glucose variability, were analyzed exclusively on days 2 and 3, during which complete 24-h data were available for all participants, as data from days 1 and 4 did not provide full 24-h recordings. The iPro2 system has been reported to demonstrate high analytical accuracy, with a mean absolute relative difference (MARD) of less than 10%, as shown in a previous validation study^51^. This analytic approach is consistent with our previous CGM-based studies using the same device^31,50^. The following CGM metrics were evaluated as previously described^31,52^: %CV and MAGE (reflecting short-term glycemic variability), MODD (reflecting intermediate-term glycemic variability), MGL, TIR, TAR, and time below range. MODD was calculated as the mean absolute differences between glucose values measured at the same time on two consecutive 24-h CGM profiles. Long-term glycemic variability was evaluated using the CV and SD of visit-to-visit HbA1c levels, while the mean HbA1c was calculated using values obtained from at least six clinical visits over 2 years immediately prior to CGM^53^ (Fig. 1). During this 2-year period, an average of 15.8 ± 2.6 HbA1c measurements were made.

### Laboratory measurements

Oxidative stress was assessed via the d-ROMs test using a specialized photometric device (F.R.E.E. System; supplied by LTD Tokyo, manufactured by Diacron International s.r.l., Grosseto, Italy), as previously reported^50^. Based on the Wismerll kinetic method, the rate of absorbance change per minute was corrected and reported in AU (U. CARR), wherein 1 U. CARR corresponds to the oxidative capacity of a 0.08 mg/dL hydrogen peroxide (H₂O₂) solution (normal range: 250–300 U. CARR). The CVs for intra- and inter-assay precision were 2.1% and 3.1%, respectively. The serum concentrations of total cholesterol, LDL-C, high-density lipoprotein cholesterol, triglycerides, and creatinine were determined using an automated analyzer (BM6070; Japan Electron Optics Laboratory, Tokyo, Japan). Plasma glucose levels were assessed using the glucose oxidase method, while HbA1c was quantified via high-performance liquid chromatography^54^.

### Measurements of SAF

The accumulation of AGEs in the skin was non-invasively evaluated by SAF using the AGE-Reader device (Diagnoptics) as previously described^23^. Autofluorescence was quantified as the ratio of the mean light intensity per nanometer within the 420–600 nm wavelength range to that within the 300–420 nm range; results were expressed in AU. Measurements were taken from the inner forearm just below the elbow crease while the patients were seated. In accordance with the manufacturer’s guidelines, three consecutive SAF readings were collected and averaged for analysis. The intra-individual CV of SAF was 5%^21^.

### End points and assessments

The primary endpoint was the association of the d-ROMs levels (a marker of oxidative stress) with short-, intermediate-, and long-term glycemic variability. The secondary endpoint was the association of SAF with short-, intermediate-, and long-term glycemic variability.

### Statistical analysis

The Shapiro–Wilk test was applied to evaluate the normality of the data distribution. Normally distributed continuous variables were expressed as the mean ± SD, while skewed data were summarized as the median (interquartile range). Depending on the distribution of the variables, simple linear associations were assessed using either Pearson’s correlation coefficient or Spearman’s rank correlation test, respectively, for normally and non-normally distributed data. Student’s t-test was employed to compare mean values between two groups, and one-way analysis of variance (ANOVA) was used for comparisons among three or more groups, since both d-ROMs and SAF demonstrated normal distribution patterns. Bonferroni adjustment was applied to correct for multiple comparisons in the analysis of d-ROMs and SAF levels among subgroups defined by retinopathy stage (i.e., none, simple, pre-proliferative, or proliferative diabetic retinopathy) and by diabetic nephropathy stage (i.e., normo-, micro-, or macroalbuminuria).

Multiple stepwise regression analyses were performed using d-ROMs levels and SAF as dependent variables. Variables that showed significant associations in univariate analyses were entered into each multivariate model. For d-ROMs, the model included age, sex, hypertension, HbA1c, MGL, GMI, %CV, MAGE, MODD, TIR, TAR, HbA1c SD, 2-year average HbA1c, HbA1c CV, diabetic nephropathy stage, thiazolidine use, and insulin use. For SAF, the model included age, duration of diabetes, hypertension, dyslipidemia, eGFR, MODD, 2-year average HbA1c, UACR, macroangiopathy, diabetic nephropathy stage, retinopathy stage, dietary treatment, insulin use, and diuretic use. The use of GLP-1 receptor agonists and SGLT2 inhibitors was also examined but neither variable showed significant correlations with d-ROMs or SAF, and they were automatically excluded by the stepwise selection procedure. Multicollinearity was evaluated using variance inflation factors (VIF), and all VIFs were < 2.0. All analyses were performed using IBM SPSS Statistics for Windows, version 22.0 (IBM Corp., Armonk, NY, USA).

## Supplementary Information

Supplementary Information 1.

Supplementary Information 2.

## Data Availability

The datasets generated and/or analyzed during the current study are not publicly available, as data sharing was not included in the informed consent. However, data are available from the corresponding author upon reasonable request and are stored in a controlled-access data repository.
