# Supplementary material for: Association of glycemic variability with oxidative stress and AGE accumulation in type 2 diabetes
Source: Sci Rep. 2025 Dec 11;16:2055. doi: 10.1038/s41598-025-31845-x (PMC12808768; doi:10.1038/s41598-025-31845-x)
Supplement: Supplementary file 1 — Supplementary Information 1. [file 41598_2025_31845_MOESM1_ESM.docx]

**Supplementary Table 1** Multiple regression analysis of independent correlates of d-ROMs

| Independent variable | Dependent variables: d-ROMs (U.CARR.) | | | | | | |
| --- | --- | --- | --- | --- | --- | --- | --- |
|  | B | SE | *β* | *t* | 95%CI | *p* value | VIF |
| Constants | 152.974 | 30.787 |  | 4.969 | 91.451-214.497 | <0.001 |  |
| MODD | 1.861 | 0.515 | 0.402 | 3.614 | 0.832-2.889 | 0.001 | 1.469 |
| Sex（Male:1、Female:2） | 44.803 | 12.886 | 0.322 | 3.477 | 19.052-70.555 | 0.001 | 1.017 |
| Nephropathy | 17.891 | 8.348 | 0.202 | 2.143 | 1.209-34.574 | 0.036 | 1.058 |
| ％CV | 2.221 | 1.072 | 0.225 | 2.073 | 0.080-4.362 | 0.042 | 1.399 |
| Sodium-glucose cotransporter 2 inhibitors use | — (excluded) | — | — | — | — | — | — |
| Glucagon-like peptide 1 receptor agonists use | — (excluded) | — | — | — | — | — | — |
| Adjusted R^2^ | 0.436 |  |  |  |  |  |  |

This table presents unstandardized (B) and standardized (*β*) regression coefficients, standard errors (SE), *t*-values, 95% confidence intervals (CIs), significance levels (*P*-values), and variance inflation factors (VIF) for each predictor.

Abbreviations:

MODD: mean of daily difference of blood glucose,

%CV: coefficient of variation
