# Supplementary material for: Association of glycemic variability with oxidative stress and AGE accumulation in type 2 diabetes
Source: Sci Rep. 2025 Dec 11;16:2055. doi: 10.1038/s41598-025-31845-x (PMC12808768; doi:10.1038/s41598-025-31845-x)
Supplement: Supplementary file 2 — Supplementary Information 2. [file 41598_2025_31845_MOESM2_ESM.docx]

**Supplementary Table 2** Multiple regression analysis of independent correlates of SAF

| Independent variable | Dependent variables: SAF (AU) | | | | | | |
| --- | --- | --- | --- | --- | --- | --- | --- |
|  | B | SE | *β* | *T* | 95%CI | *p* value | VIF |
| Constants | 2.286 | 0.182 |  | 12.581 | 1.932-2.649 | <0.001 |  |
| Duration of diabetes (years) | 0.015 | 0.007 | 0.249 | 2.241 | 0.002-0.029 | 0.029 | 1.122 |
| UACR (mg/g・Cre) | 0.000 | 0.000 | 0.233 | 2.200 | 0.000-0.000 | 0.032 | 1.021 |
| Macroangiopathy | 0.386 | 0.156 | 0.272 | 2.476 | 0.074-0.697 | 0.016 | 1.099 |
| MODD | 0.009 | 0.004 | 0.216 | 2.012 | 0.000-0.018 | 0.049 | 1.050 |
| Sodium-glucose cotransporter 2 inhibitors use | — (excluded) | — | — | — | — | — | — |
| Glucagon-like peptide 1 receptor agonists use | — (excluded) | — | — | — | — | — | — |
| Adjusted R^2^ | 0.265 |  |  |  |  |  |  |

This table presents unstandardized (B) and standardized (*β*) regression coefficients, standard errors (SE), *t*-values, 95% confidence intervals (CIs), significance levels (*P*-values), and variance inflation factors (VIF) for each predictor.

Abbreviations:

UACR: urinary albumin-to-creatinine ratio,

MODD: mean of daily difference of blood glucose
